# Immunologic Response of Unvaccinated Workers Exposed to Anthrax, Belgium

**DOI:** 10.3201/eid1510.081717

**Published:** 2009-10

**Authors:** Pierre Wattiau, Marc Govaerts, Dimitrios Frangoulidis, David Fretin, Esther Kissling, Mieke Van Hessche, Bernard China, Martine Poncin, Yvo Pirenne, Germaine Hanquet

**Affiliations:** Veterinary and Agro-chemical Research Centre, Brussels, Belgium (P. Wattiau, M. Govaerts, D. Fretin, M. Van Hessche); Bundeswehr Institute of Microbiology, Munich, Germany (D. Frangoulidis); Institute of Public Health, Brussels, Belgium (E. Kissling, B. China, G. Hanquet); Occupational Medicine PROVIKMO, Verviers, Belgium (M. Poncin); Medical Inspection, Angleur, Belgium (Y. Pirenne); European Centre for Disease Control and Prevention, Stockholm, Sweden (E. Kissling)

**Keywords:** anthrax, protective antigen, wool-sorter, ELISA, subclinical infection, bacteria, Belgium, dispatch

## Abstract

To determine immunologic reactivity to *Bacillus anthrax* antigens, we conducted serologic testing of workers in a factory that performed scouring of wool and goat hair. Of 66 workers, ≈10% had circulating antibodies or T lymphocytes that reacted with anthrax protective antigen. Individual immunity varied from undetectable to high.

Industrial anthrax, also known as woolsorter’s disease, was a serious threat in the 19th and early 20th centuries when the wool industry was flourishing. The causal agent, *Bacillus anthracis*, was brought into factories in sporulated form with the organic matter that was contaminating the animal fibers. The pathogen provoked the characteristic necrotic lesions on the skin of the wool workers (cutaneous anthrax), but it could also cause a respiratory disease through airborne transmission (inhalational anthrax). In 1950, 90% of those with the latter form died, although the proportion of deaths could be lowered to 50% with the aggressive therapy that was later used to treat the victims of the deliberate release of anthrax in the United States in 2001 (*1*,*2*).

Today, industrial processing of wool and goat hair has almost disappeared from Western industrialized countries. Cases of human anthrax have become rare in Europe (*3**,**4*), but they can sometimes result from contact with imported contaminated material (*5*,*6*). Apart from the 2001 attacks (*7*), the most recent human anthrax epidemic in the United States was reported in 1957 in a large goat hair–processing mill in Manchester, New Hampshire (*1*). In a recent study, we investigated the microbiologic flora of a Belgian factory (in operation since 1880) that processes and scours wool and goat hair from all over the world. Living anthrax spores were demonstrated in goat hair fibers, air dust, and unprocessed wastewater produced from goat hair scouring (*8*). No clinical case of anthrax was recorded among the employees of this company except for a possible cutaneous lesion, reported by a worker in 2002, the cause of which remained unconfirmed. In the current study, we investigated the immunity of the factory workers. Since none of these workers had been vaccinated against anthrax, we assumed that immunologic reactivity to anthrax antigens, if any, would very likely demonstrate exposure to *B. anthracis*.

## The Study

**Figure 1:**
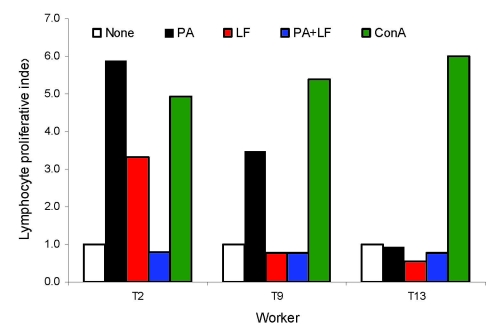
Representative examples of lymphocyte proliferation results. Proliferation was assayed by measuring ^3^H-thymidine incorporation (counts per minute [cpm]) of culture lymphocytes stimulated with different antigens and by determining the respective proliferative Indexes. The latter were calculated by dividing the cpm induced by a given antigen by the cpm induced by a negative control antigen (phosphate-buffered saline (PBS), white boxes). The proliferative index is a parameter that reflects the reactivity of a lymphocyte culture toward a given antigen. It is indicative of the cellular immunity of a person toward this antigen. The antigens used in this experiment are listed in the Table. The figure shows 3 representative culture profiles that react either with protective antigen (PA) and lethal factor (LF) (1 sample, T2), with PA only (1 sample, T9), or with none of them (41 samples, exemplified by T56). Each value is the mean of 3 independent experiments and is shown with the standard deviation (error bar). ConA, concanavalin A.

**Figure 2:**
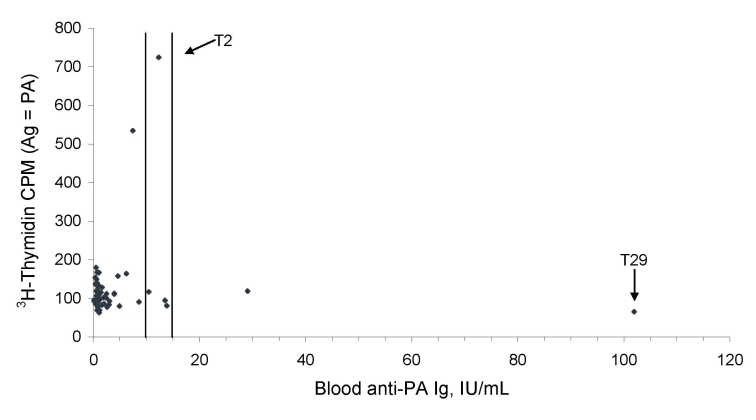
Graph showing anti–protective antigen (PA) immunoglobulin G (IgG) titers plotted against ^3^H-thymidine counts per minute (cpm) derived from PA-stimulated blood cell cultures conducted in year 2. The vertical lines define the ELISA borderline and upper thresholds (10 IU/mL and 15 IU/mL, respectively), which were defined as PA titers by the ELISA kit manufacturer, i.e., titers supposed to confer protection after vaccination. Samples testing below the borderline threshold are considered negative. T29 and T2 refer to workers whose samples had a remarkably high antibody titer or lymphocyte proliferation count, respectively.

Blood samples were obtained from 66 of the 67 factory workers, including the administrative employees. Serologic testing was carried out at 2 time points (December 2006 and December 2007) by using a commercial ELISA (Serion, Würzburg, Germany) based on plates coated with purified anthrax protective antigen (PA) (Technical Appendix). The first year, 3 workers had immunoglobulin (Ig) G titers above the threshold recommended by the manufacturer for vaccine protection (>15 IU/mL), and titers for another worker were considered borderline (10–15 IU/mL). All 4 workers had positive results by Western blot or dot blot analysis with pure recombinant anthrax PA and lethal factor (LF). One year later, 54 workers were sampled (2 were new employees). The second round of testing gave similar results, except for 3 additional borderline cases which could also be confirmed by Western blot/dot blot analysis (Table). Lymphocyte proliferation assays were performed concurrently by using fresh, heparinized, whole blood samples to evaluate the cell-mediated immunity of the workers (*9*). This technique measures the ability of lymphocytes placed in short-term tissue culture to undergo clonal proliferation when stimulated in vitro by a foreign antigen. Cell proliferation was determined by measuring the incorporation of ^3^H-thymidine into chromosomal DNA. The release of interferon-gamma (IFN-γ) in the course of lymphocyte stimulation was also measured to assess antigen-specific, cell-mediated reactivity. The antigens used here were pure recombinant PA and LF, along with positive control (concanavalin A) and negative control (phosphate buffer) stimulants. As shown in the Table, 2 cultures were positive in proliferation assays. Of these 2 cultures, 1 reacted with PA and LF, and 1 reacted with PA only. When added together, PA and LF suppressed the proliferative effect of the individual antigens, consecutive to the probable cytotoxicity induced by the 2 assembled antigens (porin + toxin). Typical examples of lymphocyte proliferation results are shown in Figure 1. The lymphocyte cultures found to be responsive to pure anthrax antigens originated from workers who had little circulating anti-PA IgG (<15 IU/mL), as tested by ELISA (Figure 2). However, their serum tested positive by Western blot analysis (Table). Moreover, supernatants of PA-stimulated lymphocyte cultures derived from the blood of these workers with positive results by determining counts per minute, were confirmed by IFN-γ release assay.

**Table:** Number of workers showing immunoreactivity against *Bacillus anthracis* antigens, as assayed by 3 different methods*

Status	Anti-PA ELISA†			Western blot/dot blot‡			L_T_ proliferation§			
	Year 1	Year 2		Year 1	Year 2		Ag = PA	Ag = LF	Ag = PA + LF	Ag = conA
Negative	62	48		–	–		52	53	54	13
Positive	3	2		4	6		2¶	1¶#	0	41
Borderline	1	4		–	–		0	0	0	0

*PA, protective antigen; LT, lymphocyte; Ag, antigen; LF, lethal factor; conA, concanavalin A. †Performed on serum samples according to the manufacturer’s instructions and thresholds (Serion, Würzburg, Germany). Note: 1 worker who tested positive at year 1 was not enrolled at year 2, and 3 workers tested negative at year 1 seroconverted to borderline at year 2. ‡Conducted only on serum samples found positive or borderline by anti-PA ELISA together with negative controls (n = 25). Dot blots were spotted with 1,000, 100, 10, and 1 ng of each purified PA and LF Ag purchased from Quadratech Ltd. (Epson, UK). Western blot antigens consisted in supernatant proteins derived from the culture of a reference *B. anthracis* strain. §Assayed on blood samples from year 2 as described earlier (9) by using a proliferative index threshold set to 3× the index of a negative control stimulant (phosphate buffer). Stimulating Ag was used at a final concentration of 4 mg/mL (PA, LF, conA). ConA was used as positive control stimulant and was purchased from Sigma (St. Louis, MO, USA). Lymphocyte cultures found activatable by PA were confirmed by quantifying IFN-γ release by ELISA, according to the manufacturer’s instructions (Pierce, Rockford, IL, USA). ¶Serum samples from these workers tested positive by both Western blot and dot blot analysis. #This culture was also stimulated by PA.

## Conclusions

Although some progress made in improving the biologic safety of the industrial processing of wool and goat hair (for example, systematic disinfection, air filtering, and protective gear for employees working in closed areas), this study shows that *B. anthracis* still poses a health risk to modern wool workers. Handling nondisinfected, raw animal fibers from areas where anthrax is endemic, such as the southern Caucasus region and the Middle East, has been and remains an at-risk activity. The presence of circulating antibodies and T lymphocytes that react with antigens expressed only by vegetative cells of *B. anthracis* in unvaccinated wool workers confirms several previous findings. First, these findings support the conclusions that anthrax spores are able to germinate into vegetative cells at the sites of exposure (skin, mucosa, respiratory tract) and cause asymptomatic infection (*10*,*11*). Second, the extent to which the human immune system responds to exposure to anthrax spores from the surrounding environment as well as the nature of this response varies tremendously from person to person. This conclusion was well exemplified by the situations of 2 workers. Results from 1 worker (T29) displayed a high IgG titer (>100 U/mL) but little or no cell-mediated reactivity. Results from the other worker (T2) showed significant lymphocyte reactivity (^3^H-thymidine counts >700 counts per minute, which corresponds to a proliferative index of 6, p>0.01), but a low IgG titer (Figure 2), which reflects reciprocal T- and B-cell responses. None of the persons whose samples tested positive by ELISA reported a past episode of anthrax (according to face-to-face interviews conducted when blood was sampled). Hence, their seroconversion most likely resulted from asymptomatic *B. anthracis* infection. One worker reported having had a skin lesion possibly compatible with cutaneous anthrax 4 years before the study. That worker’s samples tested positive by lymphocyte proliferation assay, Western blot, and dot blot, but not by anti-PA ELISA.

Notably, samples from many workers from the same factory, who had been exposed to goat hair for years in similar conditions, did not display positive serologic results when tested by ELISA. During our study, however, we noticed that serum samples from 3 workers had seroconverted from negative to partially protective (borderline) IgG titers at some point between the 2 blood samplings as determined by anti-PA ELISA. Given the long history of these workers at the company, the apparent lack of anti-PA antibodies at the first blood sampling may have been misestimated due to the high threshold defined for seropositivity by the commercial ELISA used in the study. This commercial kit is indeed primarily aimed at evaluating the efficacy of anthrax vaccination rather than at detecting antibody responses after exposure to subinfectious doses of anthrax spores (*12*). Accordingly, we noticed that of the 3 workers who seroconverted, 2 tested positive by Western blot, and 1 tested positive by dot blot when tested retrospectively at year 1. Blotting techniques seem thus more sensitive than the presently used ELISA seropositivity threshold for detecting low anti-PA antibody titers. The low sensitivity of the method used in this work to assess cell-mediated immunity (whole blood proliferation assay) may have also underestimated the actual number of workers who exhibited cell-mediated immunity against *B. anthracis*, and the results should be regarded as indicative rather than representative.

PA-based anthrax vaccines are available to protect professionally exposed people, such as the US anthrax vaccine adsorbed or the UK anthrax vaccine. These vaccines are efficient and elicit humoral responses that protect the vaccinees against toxin-associated death (*13**)*. They do require long clinical protocols and yearly boosters (*14*) and are not officially licensed in European Union member states (except the United Kingdom). According to some authors, these vaccines might not protect wool-workers efficiently against subclinical infection, spore germination, or bacteremia (*13*,*15*). Anthrax vaccines that confer long-term immunity of both the humoral and cellular type are not yet available for the general public. Vaccines with such characteristics would be highly desirable to better protect persons who work with animal products that are possibly contaminated with anthrax spores.

## Supplementary Material

Technical Appendix picrograms (10-12 g) IFN-gamma ml-1
